# Employed Caregivers’ Perceptions of Environmental Influences in Residential Dementia Care: A Qualitative Meta-Synthesis

**DOI:** 10.3390/nursrep15060183

**Published:** 2025-05-23

**Authors:** Megan Nicola Downes, Steve Hemingway, Bibha Simkhada, Nigel King, Ann-Louise Caress

**Affiliations:** 1 Department of Psychology, School of Human and Health Sciences, University of Huddersfield, Huddersfield HD1 3DH, UK; n.king@hud.ac.uk; 2 Department of Nursing, School of Human and Health Sciences, University of Huddersfield, Huddersfield HD1 3DH, UK; s.j.hemingway@hud.ac.uk (S.H.); b.d.simkhada@hud.ac.uk (B.S.); a.caress@hud.ac.uk (A.-L.C.)

**Keywords:** environment, employed caregiver perspective, qualitative research, meta-synthesis, dementia, nursing

## Abstract

**Background/Objectives**: Understanding environmental experiences in residential dementia care is crucial for enhancing care practices, training, and policy. The environment’s role in dementia care is complex. Allied health and nursing professionals must consider environmental aspects concerning care for individuals with dementia. This study investigates how employed caregivers experience and perceive environmental influences in residential dementia care. **Methods**: In September 2024, we systematically searched PubMed, CINAHL, and PsycINFO for qualitative studies, adhering to SRQR guidelines. The CASP tool was used to assess study quality, ensuring rigor and reliability in selection. The meta-synthesis is registered with Prospero: CRD42024598962. A template analysis was conducted to structure emerging themes. **Results**: Fifteen studies met the inclusion criteria. Key themes included: 1. Working Environment: Informed understandings—capturing caregivers’ perceptions of organisational structures and support. 2. Lived Environment: Stability and Clarity—highlighting the impact of relational and social dynamics on caregiving; and 3. Physical and Built Environment: Impact on Overall Care Experience—looking at the role of space and design in facilitating effective dementia care. Following this, more subthemes emerged, resulting in the final template. **Conclusions**: The key gaps included understanding the existing strengths of practice, differences in job roles, funding, the role of Employed Caregivers, and Employed Caregivers’ personhood. Emphasising evidence-based practice and clearly defining job roles may improve decision-making and collaboration. It is essential to identify funding gaps and provide clear guidelines and training to ensure equitable care and maximise the contributions of Employed Caregivers working with nurses and allied health professionals. Recognising Employed Caregivers’ personhood could enhance job satisfaction and reduce burnout. Policies should address these gaps by providing training to clarify roles and enhance skills. A supportive, person-centred workplace may improve satisfaction and care outcomes. Future research should evaluate these aspects and continue to identify the best practices.

## 1. Introduction

Dementia care poses considerable global challenges with extensive effects. The prevalence of dementia is rapidly increasing, affecting over 55 million people worldwide, and is expected to rise to 139 million by 2050 [1]. The economic impact is profound, with global costs estimated at 1.3 trillion dollars in 2019 and projected to reach 2.8 trillion by 2030 [1]. This surge in cost and demand places substantial pressure on healthcare systems and workforces.

The healthcare environment encompasses all physical, social, economic, cultural, and policy-related factors that influence the health of individuals with dementia, as well as the staff and families involved in their care. While there is a growing body of literature that explores how the environment impacts health and social care practices, there remains a gap in research specifically focused on dementia care environments. Previous studies have shown how the physical, social, and societal environment can influence older patients’ participation in healthcare [2]. Understanding the impact of the environment is key to accessing knowledge for promoting positive environments in dementia care practice [3], with nursing, allied health professionals, and Employed Caregivers playing a vital role in the delivery of effective dementia care practice.

However, the concept of the environment is complex and multifaceted, often lacking a clear definition in the literature. This complexity arises from the overlapping influences of various environmental factors. Lawton’s theory of person–environment fit proposes that an individual’s functioning is shaped by the interaction between their abilities and the demands of the surroundings [4]. As functional competencies decline in individuals with dementia, the demands of the environment become increasingly challenging to meet [5]. When applied to a dementia care context, a safe, comfortable, and enabling environment can reduce behavioural stressors, help those with dementia make the most of their abilities, and ease the pressure on caregivers [6,7].

When considering health and care professionals’ perspectives within occupational health psychology, the three-component demand-control-support (DCS) model identifies jobs with higher demands, low control, and strong support systems as being linked to improved psychological well-being and job satisfaction [7,8,9]. Further studies have found that person-centredness is connected to high job satisfaction, personal accomplishment, and the sustainability of a workforce [10,11,12].

Creating environments that facilitate better care delivery is essential, as this enhances the quality of life for residents, supports rehabilitation, and provides effective post-diagnostic care. Despite these insights, there remains a pressing need for a comprehensive evidence-based synthesis of environmental concepts within dementia care specifically. Current research often lacks a clear definition of the environment, leading to overlapping and sometimes conflicting concepts. This gap hinders the development of targeted interventions that can truly address the unique needs of those living with dementia. This review may assist in addressing issues related to the definition of environmental concepts.

Understanding the workforce’s experiences is crucial for guiding better practices and training. Employed Caregivers are essential to the effective delivery of dementia care, yet their perspectives are often underrepresented in research [13]. It is important to differentiate between informal caregivers and formal caregivers, the latter being the target population of this research. Informal caregivers are usually family members or friends providing unpaid care, while Employed Caregivers are individuals undertaking paid care work, with this study focusing on those working in residential care settings.

By examining qualitative data across studies internationally, this study aims to illustrate how Employed Caregivers experience the dementia care environment. These insights can inform future practice, research, training, and policy development. Against this background, this study addressed the under-representation of Employed Caregivers and the lack of clear definitions of the environment by exploring how caregivers experience the influence of the environment in residential dementia care practice.

Research Aim: To explore how employed caregivers experience and perceive environmental influences in residential dementia care.

## 2. Methods

This study employed a qualitative meta-synthesis approach, as outlined in the SRQR Checklist [14], to integrate data regarding Employed Caregivers’ perspectives on the influence of the environment in residential dementia care practices. This meta-synthesis is registered with Prospero: CRD42024598962. The search strategy was developed with input from an information specialist from the Computing and Library Services Team at the University of Huddersfield.

### 2.1. Research Paradigm

This research is positioned around a limited realist perspective [15]. The reason for its application in this research is centred on the belief that while an objective reality may exist, our understanding of it is inherently constrained by individual perceptions, experiences, and contextual influences. By adopting a limited realist approach, this research can acknowledge and explore the diversity of viewpoints and experiences, thereby contributing to the transferability of the synthesis. To add, it is theory-driven, which aligns with the objectives of meta-synthesis by supporting the development and refinement of theoretical foundations, which assists with explaining the occurrence of mechanisms and certain phenomena.

### 2.2. Context

The rationale for investigating this population is that Employed Caregivers are directly involved in the daily care of individuals with dementia. Their insights are key to understanding how the environment influences dementia care practices. The criterion for eligibility further indicates the contextual positioning of this meta-synthesis.

### 2.3. Eligibility Criteria

The Sample, Phenomenon of interest, Design, Evaluation, and Research type (SPIDER) was used to structure and refine the search strategy (Table 1) [16]. This framework assisted with the development of the research aim, the eligibility criteria, and the search strategy.

### 2.4. Search and Sample Strategy

This search strategy was developed with the support of an information specialist. A literature search was conducted from September to November 2024 (with the final search on 21 November 2024) using a combination of Medical Subject Headings (MeSH) terms and text words, as detailed in Appendix A. All MeSH terms and text words were organised into lines of inquiry utilising Boolean operators such as “AND” or “OR”. The search strategy was customised for each individual database. For the complete set of search terms used, refer to Appendix A.

Example search included:

PubMed: ((((((“residential home”[Text Word]) OR (“nursing home”[Text Word])) OR (“residential facilities”[MeSH Major Topic])) OR (“housing for the elderly”[MeSH Major Topic])) AND ((((“dementia”[MeSH Major Topic]) OR (“alzheimer disease”[MeSH Major Topic])) OR (dementia[Text Word])) OR (Alzheimer’s[Text Word]))) AND (((experiences[Text Word]) OR (attitudes[Text Word])) OR (perspectives[Text Word]))) AND (((((((((caregiver*[Text Word]) OR (nurs*[Text Word])) OR (matron[Text Word])) OR (therapist[Text Word])) OR (Manager[Text Word])) OR (“health professional”[Text Word])) OR (“care provider”[Text Word])) OR (“care professional”[Text Word])) OR (“caregivers”[MeSH Major Topic]))) AND (((“qualitative research”[MeSH Major Topic]) OR (interview*[Text Word])) OR (finding*[Text Word]).

Sampling continued until no new information was found in the search results. The purpose of the sampling strategy was to ensure a thorough and comprehensive understanding of the research topic.

### 2.5. Ethical Issues Pertaining to Human Subjects

This meta-synthesis did not require formal ethical review. The data synthesis from previous studies has already been subjected to ethical review and approval. The direct participant consent is not applicable. Confidentiality and data security was maintained through standard GDPR procedures.

### 2.6. Data Collection Process

An independent researcher conducted a systematic search in three databases and completed the initial screening. Data was collected and transferred into RefWorks. One researcher then screened titles and abstracts to refine the search for full-text screening.

### 2.7. Information Sources

The databases searched for this meta-synthesis were PubMed, CINAHL, and PsycINFO. While alternative databases could have been considered, these were specifically chosen to maintain a refined focus. Their specialist focus on nursing, allied health, and evidence-based practice ensured a comprehensive review of the literature. Leveraging the strengths of these databases allowed a thorough and focused analysis, with added relevancy.

### 2.8. Selection Process

Articles eligible for full-text screening were reviewed by two reviewers. They were recorded on an Excel sheet, and decisions on inclusion were made based on the eligibility criteria. Any discrepancies were resolved through discussion and the involvement of a third reviewer.

### 2.9. Data Extraction

One researcher extracted data from selected studies into a standardised Microsoft Excel sheet, including aims, country of origin, methods, themes, and key findings from 15 qualitative studies.

### 2.10. Data Analysis and Synthesis

Template Analysis [15] was used in this study to organise and interpret qualitative data systematically. Template Analysis is a well-recognised and widely used approach [15], which has previously been used successfully in structured reviews and evidence syntheses [18,19]. It provides a flexible and hierarchical structure, typically aimed at analysing complex qualitative data, with the use of *a priori* themes helping to ensure that analysis is focused and consistent. Template Analysis involves steps of familiarising with texts, identifying codes, developing a template, and applying it iteratively [15]. Two researchers critically examined the coding to ensure the credibility of the final template.

### 2.11. Trustworthiness and Risk of Bias

Continuous review and reflection ensured that inconsistencies among authors were managed, preserving synthesis integrity and transparency. Multiple reviewers helped reduce bias and increase the findings’ reliability. Two independent researchers extracted data using a standardised data extraction form, and their independent interpretations and synthesis helped reduce the risk of bias and increase reliability. An audit trail was kept throughout the process of this review to document and track progress.

### 2.12. Reflexivity

The authors recognise how their personal beliefs and biases could influence the interpretation of this study. This is because the authors of this research come from a range of diverse occupational and academic backgrounds. For example, one author comes from an occupational background working as a Senior Health Care Assistant in a dementia care unit. This position can bring an intimate knowledge of the day-to-day realities faced by caregivers. Each author has provided unique experiences and expertise, which could enhance the depth of insights presented in this review. Further backgrounds included mental health nursing, health service research, and qualitative review methods. This diversity has allowed for the exploration of the subjective realities of how participants construct meaning from their experiences. Reflexivity was addressed through maintaining a research diary, which documented any evolving thoughts and reflections. Finally, continuous engagement in regular critical discussions with the research team assisted with challenging assumptions and viewpoints. These practices helped uncover and address any potential biases between authors. The involvement of an information specialist in the development of the search strategy provided a further safeguard.

## 3. Results

### 3.1. Results of Screening

The exclusion criteria were applied through a multi-step process to ensure a rigorous and inclusive review. Initially, the SPIDER tool was employed to guide the selection and choice of qualitative studies. This assisted with defining key elements of the review and standardised the search strategy. During the screening process, the relevance and sufficiency of data were considered. Relevance was determined by whether the studies directly addressed the research aim. Studies that did not align with the focus on dementia care environments and caregiver experience were excluded. The sufficiency of data was judged based on indicators like the comprehensiveness and richness of the participants’ experiences and how well the data fit within the broader context of this study. Quality was marked on the credibility and consistency of the source, which was decided through peer review with the authors. Final assessments were made using quality assessment tools (CASP) [20].

**Figure A1:**
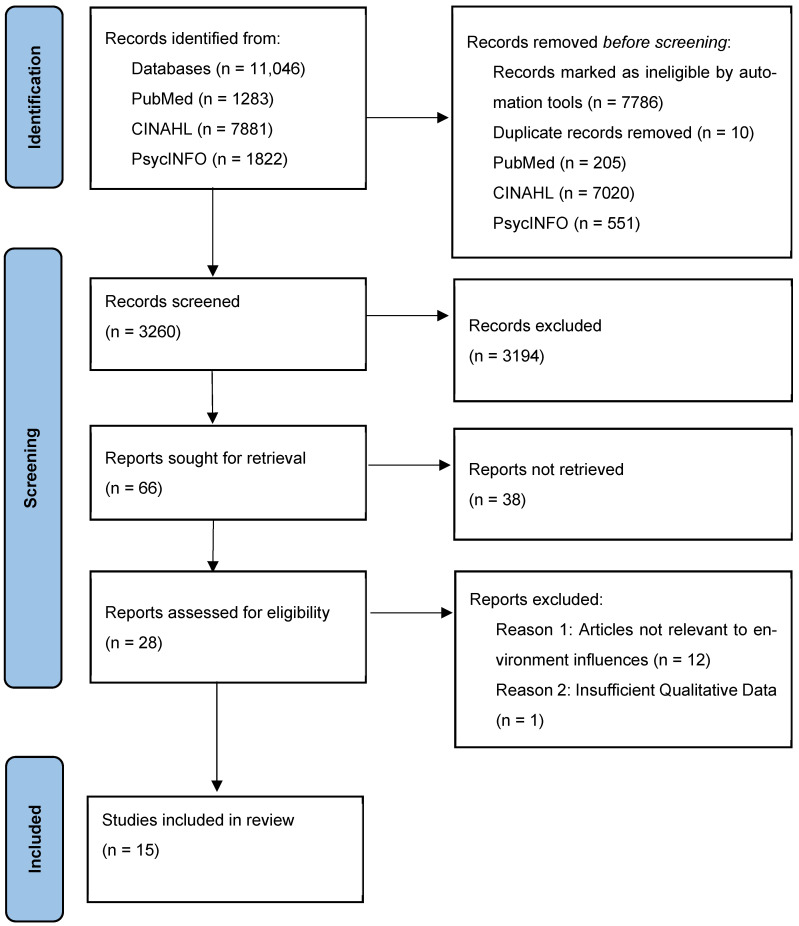
PRISMA 2020 Flow Diagram [63].

A total of 11,046 potentially relevant records were found across multiple academic databases (PubMed, CINAHL, PsycINFO) using the search strategy (please see Figure A1 in Appendix A). Automated tools assisted with the elimination of duplicates and records considered irrelevant, leading to 3260 remaining. The remaining records were reviewed based on their title and abstract, leaving 3194 excluded as they did not fit the research criteria. A total of 66 records were identified for full-text retrieval. On deeper assessment, 38 did not meet the criteria or full scope, which left 28 for a full assessment. Finally, 15 records were deemed eligible and included in the final review.

### 3.2. Units of Study

Fifteen studies were included in this meta-synthesis. These studies were conducted in 5 countries: the UK (n = 5), Canada (n = 4), the Netherlands (n = 4), Norway (n = 1), and Australia (n = 1). Most included articles utilised semi-structured interviews within their study, with some qualitative mixed methods approaches included alongside observations and focus groups (see Table 2).

In this study, the researchers solely focused on extracting and analysing qualitative data. This meant non-numerical information such as participants’ experiences, opinions, and observations. In doing so, supporting a deeper understanding of the subject through detailed, descriptive insights.

### 3.3. Quality Appraisal

The methodological quality of the included studies was assessed using the Critical Appraisal Skills Program (CASP) checklist for qualitative research [20]. This tool evaluates study quality through ten questions, rated as ‘YES’, NO’, or ‘UNCLEAR’ (see Table 3). No articles were excluded based on these scores. This was performed to ensure an unbiased and transparent analysis and recognise the value of insights from studies of varying methodological quality. [Primary weaknesses involved handling contradictory data and addressing bias and the researcher’s role. However, many studies effectively presented themes with supporting evidence and participant quotes, contributing valuable qualitative data from interviews. The CASP appraisal process involved thorough review and documentation. Question eight was examined to ensure the credibility of the studies. Overall, the articles had clear purposes, demonstrated reflexivity, and accurately represented participants’ experiences, crucial for understanding how Employed Caregivers perceive the environment in residential dementia care settings].

### 3.4. Theme Development

**Figure 1:**
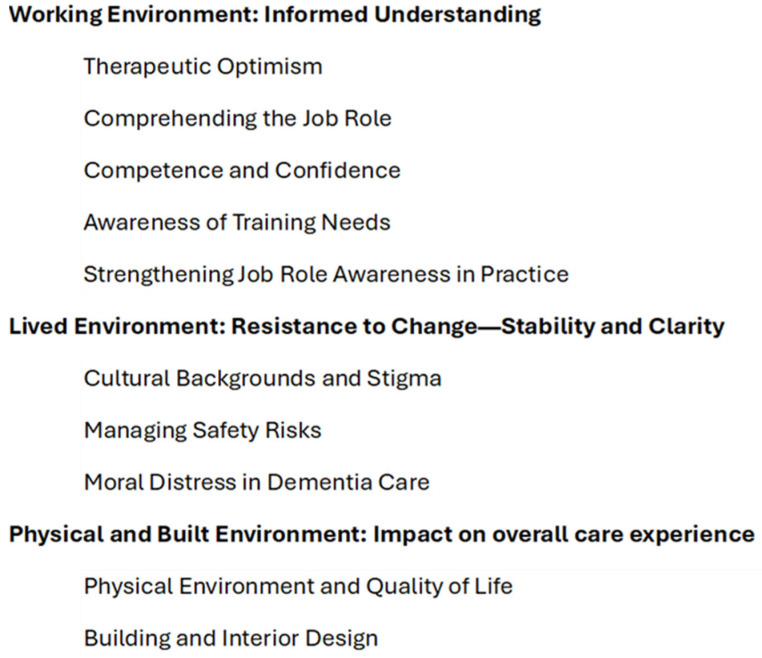
Final template.

Template Analysis incorporates both *a priori* themes, derived from the literature, and emergent themes defined in the process of developing the coding template. In our study, *a priori* themes drew on frameworks such as Lawton’s [4] and the DCS model [8]. We produced an initial version of the template based on analysing a subset of the included articles; this was then iteratively applied to further data, modified, and re-applied until a full, comprehensive version was achieved. In the final version of the template, subthemes were organised under three over-arching themes: Working Environment: Informed Understanding, Lived Environment: Resistance to Change—Stability and Clarity, and Physical and Built Environment: Impact on overall care experience. The work environment involved the settings where caregivers operated, the lived environment included the day-to-day surroundings and real-life experiences, and the physical and built environment referred to the tangible, built aspects of the spaces where dementia care is received (see Figure 1).

### 3.5. Synthesis and Interpretation

#### 3.5.1. Working Environment: Informed Understanding

All papers included highlighted the role of informed understanding in shaping the work environment. This understanding developed from a combination of different possibilities (see Table 4). The following sub-themes have illustrated how an informed understanding can resonate within the working environment for Employed Caregivers.

##### Therapeutic Optimism

Therapeutic optimism could be seen as the hope and confidence that health care providers have in their ability to achieve positive outcomes. This concept was evident in several studies, with Employed Caregivers aiming to enhance delivery and promote positive outcomes. This theme emerged through various instances such as positive interactions with residents, commitment to quality care, and maintaining presence despite other tasks. For instance, a Canadian participant expressed joy in socialising with residents, highlighting the value of genuine relationships: “*I really enjoy talking to people and socializing… I love all the interactions with the elderly; they’re very honest and real*” [21]. In the UK, a participant reflected on their dedication to providing high-quality care by questioning their efforts: “*Am I doing enough? Am I doing the right things here?*” [28]. Similarly, an Australian participant emphasised the importance of being present with residents even when managing other responsibilities: “*We are always ‘with’ them even though we had other tasks like writing in files*” [21]. These examples collectively highlighted the positive attitude Employed Caregivers have towards their work environment and their dedication to enhancing care delivery.

##### Comprehending the Job Role

Throughout studies, Employed Caregivers emphasised the need to understand their practiced roles, highlighting the importance of preparation. One participant expressed feeling unprepared due to inadequate training but noted support from colleagues [21]. Key themes included unpreparedness and lack of awareness about the role’s realities [21,32]. Studies showed a crossover in how staff view occupations in traditional versus non-traditional facilities [31]. For instance, a nurse unit manager described frustration with staff not meeting standards, leading to disillusionment: “*Normally I would try to boost them and things like that, but it’s becoming more difficult because. they’re not working to the standards that would like, so, I find that quite negative in a way because then you feel, you know, you’re not doing it, I’d like you to give more, but they are disillusioned as well (Nurse unit manager 1)*” [26]. This outlined a need for clear role definitions and consistent standards across different care settings. Additionally, food and drink were often seen as tasks rather than aspects of self-care for residents [31]. These findings underscored the need for clear role definitions and better preparation for Employed Caregivers [21], plus acknowledgment of the need to reframe these activities as integral parts of caregiving that contribute to residents’ overall well-being.

##### Competence and Confidence

Competence and confidence are crucial for Employed Caregivers, enabling high-quality care for residents. Within the articles, Employed Caregivers emphasised the importance of accepting feedback and understanding the reasons behind care approaches [24], tailoring their methods to residents’ preferences, such as using first names instead of formal titles: “*They approach her the way she wants to be approached. So, all that formal stuff like addressing residents by their surname isn’t like her. So, they address her with her first name*” (Nursing staff) [24]. Good communication within the team was also essential for building competence and confidence and adopting effective teamwork and support [24]. However, negative organisational views towards caregivers can wear down their confidence and competencies, urging the need for consistency and a positive approach in the work environment [25,26]. Employed Caregivers emphasised the importance of managing emotions in challenging situations. This emotional resilience is vital for maintaining competence and confidence in their roles [32]. Overall, competence and confidence are closely interlinked, creating a cycle of continuous improvement that benefits both Employed Caregivers and residents, strengthening the organisation and enhancing the quality of care [21].

##### Awareness of Training Needs

A proportion of participants within two of the chosen studies frequently discussed the need for more training, highlighting contradictions between training and mentorship. One participant questioned, “*How can you possibly deal with situations if you have not been shown how to?*” [32], underscoring the gap between theoretical training and practical mentorship. Training is linked to feelings of worth, job satisfaction, and capability, with Employed Caregivers suggesting the need for national regulation of the caregiver role and facilitating dementia-specific training [32]. One participant noted, “*They should be taught more about the illness before they take the role on*” [32]. Theoretical training alone was seen as insufficient for personalised care, with concerns about information retention and recall, as Employed Caregivers often remained vague about training content despite recognising its necessity. These findings could encourage a need for distinct training programs that combine theoretical knowledge with practical mentorship to further assist Employed Caregivers in the complexities of dementia care.

##### Strengthening Job Role Awareness in Practice

The need to strengthen job role awareness in dementia care practices emerged as a key theme across synthesised data. Despite Employed Caregivers recognising their training needs, barriers such as culture, time, supervision, and support systems often impeded their understanding and awareness [22,25,32]. For example, in one case, a registered nurse faced pressure to complete training on their days off, with threats of being reported to the Nursing and Midwifery Council (UK) if they did not comply: “*We’ve just not got the time to do it. Head office are saying it is not a valid excuse anymore and people are now having to come in on their day off… they are threatening staff that they’ll report them to the NMC if they don’t come in and do the paperwork*” [27] Financial constraints and heavy workloads also hindered their ability to pursue further training [27]. Clinical implications suggested that ward-based programs and reflective exercises can enhance Employed Caregivers’ competence and confidence, improving care quality [28]. A need to address systemic barriers is highlighted, while acknowledging the importance of providing adequate support to ensure Employed Caregivers can fully comprehend and perform their roles effectively.

#### 3.5.2. Lived Environment: Resistance to Change: Stability and Clarity

Employed Caregivers frequently encountered resistance to change and faced challenges in establishing stability and clarity in their daily dementia care practices. Their experiences provided insight into the underlying causes of this resistance. Furthermore, attaining stability and clarity is essential for Employed Caregivers as they endeavour to sustain the balance necessary for effective management within the context of the lived environment (see Table 5).

##### Cultural Backgrounds and Stigma

The data showed that cultural background and stigma substantially influenced dementia care environments. Personal beliefs, language barriers, and intervention approaches all played a role. For example, a First Nations Employed Caregiver in Canada struggled to adapt to a Western dementia care facility due to cultural beliefs about dementia being a natural part of life [21]. This highlighted the conflict between duty-based ethics (e.g., ensuring residents get up for their best interests) and virtue ethics (e.g., respecting residents’ wishes to refuse care). Stigma also emerged as a key issue, with Employed Caregivers often facing abuse. One noted, “*I mean, considering they are vulnerable, it’s fine. I can take the abuse. I don’t mind. I don’t care. In the end I have to do it, my dear, because otherwise I can’t work tomorrow.*” [26]. Perspectives on managing demanding situations varied by role, with home managers and senior staff accentuating immediate management rather than reporting incidents [26]. A senior health care assistant stressed the need to understand and resolve the reasons behind residents’ reactions, “*I have heard other carers say “God they are kicking off again” …There is a reason why people are reacting … You need to find out what that reason is and find out what the best way of resolving it*” (Senior Health Care Assistant) [32]. This approach requires caregivers to take an investigative approach when finding the root cause of distress, which could lead to conflicts between those who employ immediate de-escalation techniques and those advocating through investigation.

##### Managing Safety Risks

Employed Caregivers frequently face conflicting situations, impeding efforts to promote independence due to ongoing safety assessments. An Employed Caregiver mentioned that without constant safety worries, their role becomes easier and more satisfying, improving both work performance and personal satisfaction [29]. When the living environment functions cohesively, Employed Caregivers could attend to residents more effectively, fostering a person-centred and engaging approach. Risk management is a continuous cognitive process for Employed Caregivers in the daily living environment. For instance, one encouraged a resident to engage in spontaneous activity, such as raking the garden, despite the potential risks: “*I have encouraged [one of the residents] to go outside as he loves the garden, and he has grabbed a rake and for probably the last half an hour, he has been raking the gardens and that’s really settled him down*” (Care Aide) [31]. This activity helped settle the residents, which demonstrated the balance between allowing residents to live fully and managing therapeutic risks [31]. Outcomes like these can demonstrate the benefits of balancing safety concerns with promoting independence and well-being for residents. This balance could be viewed through the lens of the environmental press, where Employed Caregivers must navigate the demands of ensuring safety while promoting independence, requiring a fit between their skills and environmental challenges. Finally, active risk management strategies not only improved Employed Caregivers’ job satisfaction but also enhanced the quality of care.

##### Moral Distress in Dementia Care

Employed Caregivers often experience moral distress in their daily routine due to various pressures and ethical dilemmas. Moral distress is when one knows the ethically appropriate action yet is unable to act due to constraints like resources, time, and authority. Employed Caregivers faced the pressure to cover shifts when colleagues do not show up, leading to feelings of inadequacy, as one employed caregiver noted: “*If somebody doesn’t up on that shift then you know you really do have to stand in on that shift if nobody else will*” (Occupation: Not recorded) [28]. Additionally, demands from next of kin for treatments that may not be in the best interest of the residents create ethical predicaments, as highlighted by a nurse: “*Next of kin want intravenous treatment for every infection, but they do not see how demanding it is for the resident, as they are not present all the time*” (Nurse) [30]. Employed Caregivers often coped with distressing events by relying on peer support rather than formal stress management resources and Employed Caregivers often coped without realising it, as one participant mentioned: “*Yes, I think if we’re suffering from stress, we’re meant to ring a number for example, but I’m not going to go down that route. It’s just helping your colleague really, if your colleague’s going to get behind you just step in and help them really*” [26]. A senior care aide stated that fear of the unknown and uncertain environment contributes to moral distress “*I think from observing what carers do its fear, fear because they don’t know what is going to happen next*” [32]. Therefore, it is essential to address uncertainty in the day-to-day environment to reduce moral distress. The reliance on informal support systems to cope with stress underscores the significance of the support component of the DCS model, meaning structured and accessible stress management resources have the potential to enhance employed caregiving ability to manage job demands and reduce moral distress.

#### 3.5.3. Physical and Built Environment: Impact on Overall Care Experience

The physical environment has an impact on residents’ behaviour and Employed Caregivers’ ability to provide effective care. The following subthemes revealed how the physical environment influenced the overall care experience (see Table 6). 

##### Physical Environment and Quality of Life 

The layout of the dementia care settings can impact residents’ quality of life and Employed Caregivers’ ability to work effectively. Confined spaces with many residents can lead to irritability and behavioural issues due to high environmental pressure. As one Employed Caregiver pointed out, “*I find that the spaces of our units are quite confined and there’re a lot of people in one area like we have 29 or 30 residents on our units, in the special care unit. That has a big impact on how people behave because they just basically get on each other’s nerves sometimes*” (Staff) [29]. This confirmed how space and resident high resident density interact, advocating for spacious and well-designed environments, which reduce stress and improve resident interactions. Additionally, the physical environment affected Employed Caregivers’ ability to work effectively. For instance, a stuffy, hot environment made Employed Caregivers feel tired and less patient, which in turn impacted the residents [29]. Therefore, ensuring comfortable and conducive physical environments supports Employed Caregivers’ well-being and job satisfaction, aligning with the DCS model’s emphasis on control and support.

##### Building and Interior Design

The design of spaces also affected the residents’ experience. Institutional-looking environments, like hospital corridors, can be unwelcoming [35]. Conversely, home environments with easy access for residents, large windows, and bright lights can improve job satisfaction and care quality [29]. Employed Caregivers appreciated environments where proximity to residents is carefully considered. For example, “*What I find about my working environment is the home-like feel and I can reach my residents quickly. I don’t need to walk miles to get to them. Also, what I find is I don’t know who enjoys the place more, I do or the residents, just because of the comfort, the big windows, the bright lights. It’s such a nice environment to work. When I feel good, I can be more beneficial to give care to the residents*” (staff) [29]. This positive environment not only improved their own well-being but also allowed them to provide better care to residents. Additionally, smaller-scale living facilities are viewed more positively by Employed Caregivers due to the increased opportunity for interaction with residents [33,34]. Although studies indicated how research should explore how physical environmental features and quality care practices contribute to residents’ mealtime experiences [23].

## 4. Discussion

This review has systematically identified and explored the findings of 15 qualitative studies to understand how Employed Caregivers perceive the influence of the environment in residential dementia care settings. A template analysis has indicated that the environment impacted opportunities for informed understandings in the work environment, stability and clarity of the lived environment, as well as the physical and built environment’s influence on the overall care experience. The findings revealed how environmental complexity can either create obstacles to quality care or support positive experiences and good practice within the context of dementia care. To add, Template A explored how Employed Caregivers’ perceptions of the working environment are central to understanding their roles, building competence, and staff confidence. Also, strengthening their role awareness in practice.

Consistent with previous research, which addresses the need to improve specialised dementia care knowledge and skills, due to inadequate knowledge being found to directly impact person-centred care [36,37]. Prior research has established that teaching alone is not sufficient for dementia care training, suggesting how training interventions work best when practical support is provided [38]. Moreover, data taken from a systematic review assessed the impact of dementia care educational programs on nursing home staff preparedness, finding primary outcomes in relation level of staff knowledge, attitudes, competence, and self-efficacy, all linking to the delivery of dementia care [39].

Furthermore, dementia care managers’ qualifications have been found to enhance nurse competencies to care for people with dementia in Germany [40]. In Australia, current guidelines and recommendations for the management of people with dementia aim to produce evidence-based clinical pathways to be used in Australian residential care facilities [41]. Across studies internationally, communication skills training in dementia care has been found to improve the quality of life and well-being for people with dementia and facilitate positive interactions in various care settings [42]. Further studies are calling for more organisational support to support educational interventions in dementia care and palliative care settings [43]. Finally, studies have acknowledged how across high-income countries there is an increase in awareness of the demands of dementia care, though the limits remain within the implementation of educational programs [44].

In support of Template B, risk management literature emphasises the importance of safety culture in improving care quality, through strategies aimed at minimising risk and preventing harm [45,46]. Whereas the culture of safety has been known to ignore the social and psychological well-being of people with dementia and therefore compromise the notion of person-centred care [47]. The daily circumstances experienced by Employed Caregivers showed their feelings towards change and their ability to achieve stability and clarity. The culture of care settings is essential for Employed Caregivers to cope with daily demands, relying on organisational dynamics and the attitudes and beliefs of the staff. Fitting with template b, the culture within allied health and nursing care can be explained through three levels: visible manifestations, shared ways of thinking, and deeper shared assumptions, all of which contribute to sustained current patterns of clinical practice [48]. Similarly, change initiatives within the dementia care context have related results, which have addressed the difficulties and resistance, yet have found that those who took part achieved a sense of pride and purpose [49].

Additionally, the physical environment in this meta-synthesis was important for the quality of life of the overall care experience, and elements of the built environment, such as lighting and interior design, determined the atmosphere for Employed Caregivers. Previous literature has explored the effectiveness of environmental personalisation on older people’s health outcomes and found that personalisation of the environment improved health outcomes significantly; however, more research is needed that is specific to dementia care and more empirical data which focuses on evaluating what interventions and dementia friendly renovations work best [50].

This review covers literature spanning a period of 15 years and includes international perspectives. This is structural to this research as it highlights the extent to which experiences are repeated and how deeply rooted and widespread these experiences are among Employed Caregivers. For example, the lived environment is of crucial importance due to the emotional ties associated with it. The repetition of these experiences also underlined the impact of the sub-themes presented, such as cultural backgrounds and stigmatisation. Understanding cultural values within dementia care has been previously studied; however, studies found limited evidence on the impact of cultural values on the provision of person-centred dementia care [51]. Studies have found a lack of research on interventions that address organisational culture in older people’s care settings [52].

Therefore, resistance to change and stability, and clarity are key issues that comprehend the reasons for delaying improvements in the dementia care environment, as the environment is composed of Employed Caregivers who are all essential to dementia care. Resistance to change in the lived environment is consistent with previous findings that have explored the impact of a dementia-friendly ward environment with qualified nurses, finding that staff were unable to cope with the new daily changes due to main concerns which focused on staffing levels, time management, training needs, and cultural resistance [53]. Finally, cultural influences have an impact on caregiving practices through communication styles, family dynamics, stigma, and beliefs. These environmental influences can impact the delivery of person-centred care, both positively and negatively. As an example, cultural beliefs around illness can impact how care is provided. Especially in relation to help-seeking in mental health and cultural taboos can restrict who can provide care, all impacting the quality and consistency of care. Therefore, further findings have addressed the need for culturally inclusive programs to encourage organisations to operate in a way that demonstrates cultural competence. This is important because cultural backgrounds are widely recognised as a barrier towards workforce retention [54,55,56].

## 5. Implications for Practice and Research

Funding considerably impacts Employed Caregivers’ experiences, influencing all identified themes despite limited data. Budget cuts lead to staff shortages, increased caregiver stress, and compromised care delivery, as caregivers manage multiple tasks, reducing care quality. Employed Caregivers are essential in dementia care, providing the most face-to-face care. Prior research found that Employed Caregivers receive a small fraction of the training budget, and despite training, they often feel unprepared to provide optimal care [12]. Future research could explore broader implications of funding impacts on caregivers to develop strategies that mitigate negative effects.

The psychological well-being of care assistants in dementia care is a key concern, with subthemes like moral distress and stigma highlighting their demanding work. The caregiver–care recipient relationship is linked to anxiety and depression levels [57], impacting the quality of care [58,59]. Research should aim to enhance role understanding and clarity, improving confidence, competence, and training needs. This could foster a more informed and optimistic caregiving environment, benefiting both caregivers and residents. As key figures in care coordination, nurses and allied health professionals play a crucial role in guiding care assistants, enhancing training, and encouraging best practices. Therefore, current education and professional development frameworks should reflect the number of Employed Caregivers and the scale of their caregiving responsibilities.

Recognising the personhood of Employed Caregivers is essential, as overlooking it can hinder individual-centred care and reduce self-efficacy, which leads to negative outcomes. By mirroring the values of person-centred care [60], more extensive research into Employed Caregivers’ personhood could enhance informed understanding in the dementia care environment, enhancing the care provision and staff well-being. Empathising with the emotional and psychological needs of Employed Caregivers is central to maintaining high-quality care (see Table 7).

Understanding job role differences in dementia care is fundamental, as role conflicts can negatively impact team morale and caregiving quality. Research shows that shared communication and IT systems support integration and continuity of care [61]. However, more research is required to develop strategies to mitigate conflict and promote a cohesive team. While strengths of practice in dementia care are understudied, evidence suggests a connection between the organisational environment and person-centred care delivery [62]. Future research should focus on emerging strengths of current practice models, new perspectives, and emerging ideas to overcome barriers to person-centred care. Identifying effective practices can help develop robust care models and improve overall care quality.

## 6. Strengths and Limitations

Determining the difference between roles like care aide and health care assistant was challenging, so terminology from included studies was used, along with the term Employed Caregivers. Theme compression led to the loss of some caregiving experiences in dementia care. Although the three types of environments captured by overarching themes (working environment, lived environment, and physical and built environment) proved a useful lens for review, there were sometimes overlaps between them. Especially, between the ‘work’ and ‘lived’ environments. This reflected the interconnectedness of these facets of the environment but also highlighted the need for further research to strengthen the distinct definitions. The interconnected nature of these themes demonstrates the complexity of how various elements of the environment influence one another. The review method provided a comprehensive analysis of how the environment is experienced by caregivers. The systematic meta-synthesis included an extensive search of three dementia care databases. While incorporating an international perspective presents demographic and contextual issues, it unites commonly shared views, highlighting the environment’s influence on dementia care. The review adhered to specific reporting criteria and quality assessment tools to ensure quality. Although relevant literature and practical implications outside this meta-synthesis could have been overlooked, the selected studies followed specific inclusion/exclusion methods to reveal caregivers’ perspectives of the environment in dementia care.

## 7. Conclusions

In conclusion, this meta-synthesis highlighted notable gaps in the literature, particularly in understanding the personhood of Employed Caregivers, the long-term implications of person-centred care practices, and the impact of funding on employee performance and job satisfaction in dementia care. Future research should focus on these areas, as well as the unique challenges faced by Employed Caregivers and the nuanced understanding of job role differences within interdisciplinary teams. Furthermore, there is a need to address systemic barriers within the work environment and facilitate new ways to improve employee well-being and organisational efficiency. Addressing these key areas through targeted and extended research and practice developments could enhance the dementia care workforce, resulting in improved outcomes for both residents and Employed Caregivers. 

## Figures and Tables

**Table 1:** The SPIDER Framework.

Framework	Inclusion Criteria	Exclusion Criteria
S	Nursing and allied health and social care employees in residential dementia care homes, such as unit managers, health care assistants, nursing assistants, and nurses. Inclusive of both male and female participants aged 18+ with at least 12+ months experience. There were no restrictions on the country of origin to ensure cultural sensitivity and applicability across diverse settings, identifying gaps in research by considering countries with national dementia plans [17].	Retired staff Less than 12 months experience
PI	Employed Caregivers’ perceptions and experiences of the environment’s influence on dementia care practice.	Exclusion criteria involved studies that did not report experiences related to the environment in dementia care.
D	This study included qualitative approaches such as interview data, focusing on peer-reviewed, empirical studies published between 2009 and 2024. This time frame accounts for significant socio-economic events like the financial crisis of 2007–2009 and the COVID-19 pandemic, which impacted dementia care environments. These socio-economic shifts influenced funding, resources, and care practices. Therefore, urging adaptive and sustainable care models, making studies from this period particularly relevant. Research prior to 2009 may not reflect these key shifts.	Statistical data
E	Qualitative Data and Findings Interview data	
R	Qualitative Research	Solely Quantitative Studies

**Table 2:** Key features and characteristics of 15 included studies.

Author and Year of Publication	Country	Study Aims	Methods of Data Collection and Analysis	Themes Found	Key Findings
Ref. [21] Booi, L., Sixsmith, J., Chaudhury, H., O’Connor, D., Young, M., & Sixsmith, A. (2021).	Canada	To gain insight into the everyday realities facing care aides working in long-term residential care and how they perceive society.	A qualitative ethnographic case study Semi-structured interviews Thematic Analysis	Lack of training Support Appreciation care aides felt about their role	Highlights societal ageism, gendered body care work, and the tension between relational connections needing time and economic profit.
Ref. [22] Brannelly, T., Gilmour, J. A., O’Reilly, H., Leighton, M., & Woodford, A. (2019).	UK	To explore the experiences of care support and family members on the impact of a new care approach in a specialised unit as it shifted to an inclusive model.	Qualitative thematic approach Focus Groups Thematic Analysis	Personalised care for people with dementia. Family involvement Continuing to care Staff competence Confidence to care	Participants identified effective working methods that benefited both staff and families and reported improved well-being for individuals with dementia in the unit.
Ref. [23] Chaudhury, H., Hung, L., Rust, T., & Wu, S. (2016).	Canada	To examine the impact of environmental renovations in dining spaces of a long-term care facility on residents’ mealtime experience and staff practice in two care units.	Ethnographic observations. Staff Survey Observational Data	Autonomy Personal control Comfort of homelike environment Conducive to social interaction increased personal support. effective teamwork	Physical environmental renovations yield positive outcomes for both residents and staff, additionally facilitating improved person-centred care for all involved.
Ref. [24] De Boer, B., Hamers, J. P., Zwakhalen, S. M., Tan, F. E., Beerens, H. C., & Verbeek, H. (2019).	Netherlands	To explore from the perspectives of the informal caregivers of people with dementia, the positive and negative experiences with diverse types of nursing homes.	Semi-structured interviews Exploratory research design Thematic Analysis Phenomenological approach	Experiences with the care environment. The physical environment and atmosphere Activities Person-centred care Communication Staff	The experiences of caregivers in nursing homes vary based on the specific nursing home and the individual nursing staff.
Ref. [25] Garcia, L. J., Hébert, M., Kozak, J., et al. (2012)	Canada	To explore the perceptions of family and staff members on the potential contribution of environmental factors that influence disruptive behaviours and quality of life of residents with dementia living in long-term care homes.	Qualitative Focus groups	Facility, staffing, and resident factors to consider when creating optimal environments. Human environments were seen as more important than physical environments, and flexibility was judged essential. Noise was identified as one of the most crucial factors influencing behaviour and quality of life of residents	Mnemonic for key environmental factors (CAREFUL): consistency, approach, staff-to-resident ratio, environmental design, flexibility, understanding, and noise level.
Ref. [26] Kadri, A., Rapaport, P., Livingston, G., Cooper, C., Robertson, S., & Higgs, P. (2018).	UK	To explore how the personhood of paid carers of people with dementia can be understood by focussing on the views and experiences of care home staff.	Secondary Qualitative Analysis Interviews	Delivering PCC: issues related to dementia. Issues relating to organisation. Identity of care staff. Views of care role.	Oversight of care staff can turn care work into mere tasks, lower self-efficacy, and obstruct individual-centred care. Many care staff are not recognised individually by their employers, and the moral aspects of formal care work often go unacknowledged.
Ref. [27] Killett, A., Burns, D., Kelly, F., Brooker, D., Bowes, A., La Fontaine, J., Latham, I., Wilson, M., & O’neill, M. (2014).	UK	What are the individual circumstances, organisational cultures, and practices most likely to encourage, or inhibit, the provision of high-quality care for older people living in residential and nursing homes?	Interviews Observations	7 values, attitudes, and behaviours named.	Seven inter-related cultural elements were key to the importance of care quality.
Ref. [28] Law, K., Patterson, T. G., & Muers, J. (2017).	UK	To explore the experiences of health care assistants working with people with dementia in UK residential care homes.	IPA Semi-structured interviews	The importance of relationships Something special about the role Personal commitment to the job The other side of caring	Staff should build strong, supportive relationships in their roles and have opportunities to explore their emotional responses to minimise negative effects on care provision.
Ref. [29] Lee, S. Y., Chaudhury, H., & Hung, L. (2014).	Canada	To explored staff perceptions of the role of physical environment in dementia care facilities in affecting resident’s behaviours and staff care practice.	Focus groups	A supportive physical environment contributes positively to both quality of staff care interaction and residents’ quality of life Unsupportive physical environments contribute negatively to residents’ quality of life and thereby make the work of staff more challenging	A staff collective view that comfort, familiarity, and an organised space were essential therapeutic resources for the well-being of residents.
Ref. [30] Midtbust, M. H., Gjengedal, E., & Alnes, R. E. (2022).	Norway	To gain a deeper understanding of nursing staff members’ experiences of moral distress while providing palliative care for residents with severe dementia in long-term care facilities.	Qualitative descriptive design Thematic Analysis In-depth interviews	Experiences of moral distress in two types: Those in which nursing staff members felt pressured to provide futile end-of-life treatment. Those who felt that they had been prevented from providing necessary care and treatment	Moral distress often arises from institutional constraints like time limits, challenging priorities, and value conflicts.
Ref. [31] Richards, K., D’Cruz, R., Harman, S., & Stagnitti, K. (2015).	Australia	To compare these two environments in rural Australia, and their. Influence on residents’ occupational engagement.	The residential environment impact survey Observations Interviews Thematic Analysis	Comfortable environment Roles and responsibilities Getting to know the resident. More stimulation can elicit increased engagement. The home-like experience. Environmental layout.	Research shows that non-traditional dementia facilities enhance occupational involvement, leading to positive outcomes.
Ref. [32] Talbot, R., & Brewer, G. (2015).	UK	To address the paucity of research in this area, the present study examined care assistant experiences of dementia care in British long-term residential and nursing environments.	Semi-structured interviews IPA	Psychological wellbeing of the care assistant. Barriers to effective dementia care The dementia reality Organisational issues within the care environment	The benefits of the care dyad were noted, and the organisation’s role in causing burnout and depersonalisation was emphasised.
Ref. [33] Van Zadelhoff, E., Verbeek, H., Widdershoven, G., van Rossum, E., & Abma, T. (2011).	Netherlands	To investigate experiences of residents, their family caregivers, and nursing staff in group living homes for older people with dementia and their perception of the care process.	Naturalist design Systematic participatory observations Semi-structured interviews	Residents Family Nursing Staff	Group living homes provide opportunities for individual care, meeting residents’ needs with increased attentiveness. This aligns with Tronto’s care ethical model phases of caring about and receiving care. However, tensions arise in taking responsibility and performing self-care, as not all residents and family members can or want to do so.
Ref. [34] Verbeek, H., Zwakhalen, S. M., van Rossum, E., Kempen, G. I., & Hamers, J. P. (2011).	Netherlands	To gain an in-depth insight into the experiences of family caregivers and nursing staff with small-scale living facilities	Interviews Survey questionnaire	Family Caregivers Positive aspects of small-scale living facilities Experiences with care service delivery Homeliness in small-scall facilities. Nursing Staff Skills Negative aspects of small-scale facilities Positive aspects of working in a small-scale living facility. Negative aspects of working in a small-scale living facility.	Both family caregivers and staff reported positive experiences with small-scale living facilities, highlighting personal attention, resident involvement, and autonomy. However, barriers include nursing staff working alone much of the day. Family caregivers in these facilities were more satisfied than those in regular wards.
Ref. [35] Van Hoof, J., Verbeek, H., Janssen, B. M., Eijkelenboom, A., Molony, S. L., Felix, E., Nieboer, K. A., Zwerts-Verhelst, E. L., Sijstermans, J. J., & Wouters, E. J. (2016).	Netherlands	To investigate the factors influencing the sense of home of older adults living in the nursing home from the perspective of residents, relatives, and care professionals.	Focus groups. Interviews Photography as a supportive tool	Building and interior design Eating and drinking Autonomy and control Involvement of residents Engagement with others and activities Quality of care Connection with nature and outdoors Coping Organisation and facilitative of care To matter	The sense of home for nursing home residents is influenced by building design, eating and drinking, autonomy and control, involvement of relatives and others, activities, and the quality of care.

**Table 3:** Quality appraisal.

Author and Year of Publication	Q1	Q2	Q3	Q4	Q5	Q6	Q7	Q8	Q9	Q10	TOTAL
Ref. [21] Booi, L., Sixsmith, J., Chaudhury, H., O’Connor, D., Young, M., & Sixsmith, A. (2021).	Y	Y	Y	Y	Y	Y	Y	-	Y	Y	9
Ref. [22] Brannelly, T., Gilmour, J. A., O’reilly, H., Leighton, M., & Woodford, A. (2019).	Y	Y	Y	Y	Y	Y	-	-	Y	Y	8
Ref. [23] Chaudhury, H., Hung, L., Rust, T., & Wu, S. (2016).	Y	Y	Y	Y	Y	Y	Y	Y	Y	Y	10
Ref. [24] De Boer, B., Verbeek, H., Zwakhalen, S. M. G., & Hamers, J. P. H. (2019).	Y	Y	Y	Y	Y	-	-	-	Y	Y	7
Ref. [25] Garcia, L. J., Hébert, M., Kozak, J., Sénécal, I., Slaughter, S. E., Aminzadeh, F., Dalziel, W., Charles, J., & Eliasziw, M. (2012)	Y	Y	Y	Y	-	-	-	-	Y	Y	6
Ref. [26] Kadri, A., Rapaport, P., Livingston, G., Cooper, C., Robertson, S., & Higgs, P. (2018)	Y	Y	Y	Y	-	-	Y	Y	Y	Y	8
Ref. [27] Killett, A., Burns, D., Kelly, F., Brooker, D., Bowes, A., La Fontaine, J., Latham, I., Wilson, M., & O’Neill, M. A. (2014).	Y	Y	Y	Y	Y	Y	Y	Y	Y	Y	10
Ref. [28] Law, K., Patterson, T. G., & Muers, J. (2017)	Y	Y	Y	Y	Y	Y	Y	Y	Y	Y	10
Ref. [29] Lee, S. Y., Chaudhury, H., & Hung, L. (2014)	Y	Y	Y	Y	Y	Y	Y	Y	Y	Y	10
Ref. [30] Midtbust, M. H., Gjengedal, E., & Alnes, R. E. (2022)	Y	Y	Y	Y	Y	Y	Y	Y	Y	Y	10
Ref. [31] Richards, K., D' Cruz, R., Harman, S., & Stagnitti, K. (2015)	Y	Y	Y	Y	Y	Y	Y	Y	Y	Y	10
Ref. [32] Talbot, R., & Brewer, G. (2015)	Y	Y	Y	Y	Y	Y	Y	Y	Y	Y	10
Ref. [33] Van Zadelhoff, E., Verbeek, H., Widdershoven, G., van Rossum, E., & Abma, T. (2011)	Y	Y	Y	Y	Y	Y	Y	-	Y	Y	9
Ref. [34] Verbeek, H., Zwakhalen, S. M. G., van Rossum, E., Kempen, G. I. J. M., & Hamers, J. P. H. (2011)	Y	Y	Y	Y	Y	Y	Y	Y	Y	Y	10
Ref. [35] Van Hoof, J., Verbeek, H., Janssen, B. M., Eijkelenboom, A., Molony, S. L., Felix, E., Nieboer, K. A., Zwerts-Verhelst, E. L. M., Sijstermans, J. J. W. M., & Wouters, E. J. M. (2016).	Y	Y	Y	Y	Y	Y	Y	Y	Y	-	9

KEY: Y = YES, N = NO, - = NOT CLEAR.

**Table 4:** Template A.

Working Environment: Informed Understanding The theme of the Working Environment: informed understanding has embraced and combined important practices/concepts in healthcare such as informed decision making and informed consent, etc. Working Environment: Informed understanding has a deeper meaning in that it values the idea that information is central to Employed Caregivers, with the level of understanding being the essential element required for a positive outcome in the work environment. For example, when an informed understanding is present, the following subthemes can be successfully implemented in practice.
**Therapeutic Optimism**
**Comprehending the Job Role**
**Competence and Confidence**
**Awareness of Training Needs**
**Strengthening Job Role Awareness in Practice**

**Table 5:** Template B.

Lived Environment: Resistance to Change: Stability and Clarity Resistance to change in daily life can be attributed to certain key experiences. Employed Caregivers need for stability and clarity is influenced by the following themes found in their daily encounters. These themes contributed to difficulties in maintaining stability and clarity in the lived environment. Regular occurrences of the following subthemes increased environmental pressure, which leads to stagnation in some areas and impacts the capacity for stability and clarity.
**Cultural Backgrounds and Stigma**
**Managing Safety Risks**
**Moral Distress in Dementia Care**

**Table 6:** Template C.

Physical and Built Environment: Impact on overall care experience Employed Caregivers saw how the physical environment affected their work performance, which in turn influenced the overall care experience. A well-designed physical and built environment not only enhanced quality of life for residents but supported Employed Caregivers in performing their duties more effectively. Consequently, the design and layout of a room changed both the quality of care provided and the well-being of the Employed Caregivers.
**Physical Environment and Quality of Life**
**Building and Interior Design**

**Table 7:** Recommendations for Stakeholders.

**1.** **Immersive Communication Training:** Simulations to immerse Employed Caregivers in realistic scenarios, enhancing their communication through interactive, hands-on experiences. **2.** **Empathy-Driven Support Systems:** Develop support platforms that provide real-time emotional and professional guidance to Employed Caregivers, these may provide personalised stress management resources and peer-to-peer connections. **3.** **Curricular development in Cultural Awareness and Inclusivity:** Improving knowledge on care of diverse residents and creating environments that respect cultural traditions and working practices. **4.** **Enhanced Health Care Assistant Training:** Structured training programs that align with the scale of their responsibilities. Nurses and Allied Health Professionals could lead initiatives that provide education, recognition, and integration into workforce planning.

## Data Availability

The data presented in the study are available on request from the corresponding authors.

## Public Involvement Statement

No public involvement in any aspect of this research. This was justified for a literature-based study, focusing on Employed Caregivers.

## Guidelines and Standards Statement

This manuscript was drafted following the Standards for Reporting Qualitative Research (SRQR) [14]. A complete list of reporting guidelines can be accessed via: https://journals.lww.com/academicmedicine/fulltext/2014/09000/standards_for_reporting_qualitative_research__a.21.aspx (accessed on 5 May 2025).

## Appendix A

**Data Base**	**PubMed**	**CINALH**	**PsycINFO**
Search Terms	((((((“residential home”[Text Word]) OR (“nursing home”[Text Word])) OR (“residential facilities”[MeSH Major Topic])) OR (“housing for the elderly”[MeSH Major Topic])) AND ((((“dementia”[MeSH Major Topic]) OR (“alzheimer disease”[MeSH Major Topic])) OR (dementia[Text Word])) OR (Alzheimer’s[Text Word]))) AND (((experiences[Text Word]) OR (attitudes[Text Word])) OR (perspectives[Text Word]))) AND (((((((((caregiver*[Text Word]) OR (nurs*[Text Word])) OR (matron[Text Word])) OR (therapist[Text Word])) OR (Manager[Text Word])) OR (“health professional”[Text Word])) OR (“care provider”[Text Word])) OR (“care professional”[Text Word])) OR (“caregivers”[MeSH Major Topic]))) AND (((“qualitative research”[MeSH Major Topic]) OR (interview*[Text Word])) OR (finding*[Text Word])	((MH “Nursing Homes/EI/OG/PF/ST”) OR (MH “Home Health Aides/OG/PF/EV/EI”) OR (MH “Nursing Home Patients/PF/EI”) OR “(“residential home”) OR (“nursing home) OR (“residential facilities”) OR (“housing for the elderly”)) AND ((“dementia”) OR (“alzheimer disease”) OR (dementia) OR (Alzheimer’s)) AND ((experiences) OR (attitudes) OR (perspectives)) AND ((caregiver*) OR (nurs*) OR (matron) OR (therapist) OR (Manager) OR (“health professional”) OR (“care provider”) OR (“care professional”) OR (“caregivers”)) AND ((“qualitative research”) OR (interview*) OR (finding*)) AND ((MH “Home Care Equipment and Supplies/OG/EI/ST”) OR (MH “Residential Care”) OR (MH “Caregiver Attitudes”) OR (MH “Housing for Older Persons”) OR (MH “Nursing Home Design and Construction/PF/ES/EI”) OR (MH “Residential Facilities”) OR (MH “Professional-Client Relations”) OR (MH “Professional Practice, Research-Based”) OR (MH “Caregiver Burden”) OR (MH “Home Safety”) OR (MH “Caregiver Emotional Health (Iowa NOC)”) OR (MH “Caregiver Physical Health (Iowa NOC)”) OR (MH “Health Care Delivery”))	(residential home* OR nursing home* OR residential facilit* OR “housing for the elderly” OR Care home* OR acute care unit*) AND (dementia OR Alzheimer*) AND (caregiver* OR nurs* OR matron* OR therapist* OR Manager* OR “health professional*” OR “healthcare professional*” OR “care provider*” OR “care professional*” OR “caregiver*” OR Care Assistant*) AND (experience* OR attitude* OR perspective* OR view*) AND (qualitative OR interview*) AND la.exact(“English”) AND PEER(yes)
